# Differential tumor infiltration by T-cells characterizes intrinsic molecular subtypes in breast cancer

**DOI:** 10.1186/s12967-016-0983-9

**Published:** 2016-07-29

**Authors:** M. Miyan, J. Schmidt-Mende, R. Kiessling, I. Poschke, J. de Boniface

**Affiliations:** 1 Department of Molecular Medicine and Surgery, Karolinska Institutet, Stockholm, Sweden; 2 Department of Oncology and Pathology, Cancer Center Karolinska, Karolinska Institutet and Karolinska University Hospital, Stockholm, Sweden; 3 Department of Pathology, Karolinska University Hospital, Stockholm, Sweden; 4 Division of Molecular Oncology of Gastrointestinal Tumors, German Cancer Research Center, Heidelberg, Germany; 5 Department of Breast and Endocrine Surgery, P9:03, Karolinska University Hospital, 17176 Stockholm, Sweden

**Keywords:** Breast neoplasms, Cytotoxic T-lymphocytes, Regulatory T-lymphocytes, Tumor-infiltrating lymphocytes, Molecular subtypes

## Abstract

**Background:**

Molecular subtypes of breast cancer and presence of tumor-infiltrating immune cells have both been implicated as important predictive and prognostic factors for improved risk stratification and treatment individualization of breast cancer patients. Their association, however, has not been studied in detail. The aim of this study was to evaluate the expression of the T cell markers CD8, FoxP3, CD3 and ζ-chain in molecular subtypes of the invasive margin and tumor center of breast cancer and corresponding sentinel nodes and to deduct prognostic information from these findings.

**Methods:**

Tumor and sentinel node sections from 177 patients with primary, invasive, unilateral early-stage breast cancer were stained by immunohistochemistry and T-cell phenotypes quantified manually. Clinical data were collected from medical records.

**Results:**

The degree of T-cell infiltration and expression of all markers differed significantly among the molecular subtypes, being highest in non-luminal, more aggressive tumors: more T-cell infiltration and higher expression of all markers were associated with hormone receptor negativity, higher proliferation and higher histological grades, but also with larger tumor size. Basal-like tumors, and most remarkably their tumor centers, hosted the highest number of FoxP3+ T-cells with an unfavorable ratio to cytotoxic CD8+ T-cells. T-cell infiltration was generally higher in the invasive margin than the tumor center. A scoring system based on densities of CD3 and CD8 could significantly separate molecular subtypes (p < 0.001).

**Conclusions:**

Thus, immunological patterns with functional implications within each subtype are associated with prognostic factors. These findings should be further validated in studies using larger patient populations and longer follow-up.

**Electronic supplementary material:**

The online version of this article (doi:10.1186/s12967-016-0983-9) contains supplementary material, which is available to authorized users.

## Background

Breast cancer is the most common cancer among women, with an annual incidence of more than 1.6 million cases worldwide which is expected to increase further [1]. The past decades have seen significant improvements in patient survival, likely due to increased awareness, earlier detection through screening programs and advances in treatment [2]. Further improvements are dependent on precise classification for prognostication and prediction, and for the adequate choice of individualized treatment alternatives.

In order to modify and modernize breast cancer classification, Perou et al. [3] proposed five genetically distinct groups based on gene expression microarrays. These ‘molecular subtypes’ have repeatedly been validated since and shown to have accurate prognostic potential [4]. There are commercially available multigene signature assays for subdivision of tumors into these molecular subtypes, however, estrogen and progesterone hormone receptors, the growth factor receptor Her2/Neu and the proliferation marker Ki-67 are acknowledged as clinical surrogate markers [5]. These possess the advantage of being widely used in routine practice, affordable and available [6].

Apart from the above-mentioned molecular subtypes, immunological tumor-host interactions have received increasing attention as a prognostic and predictive tool [7, 8]. Immune evasion, i.e. the ability of the tumor to avoid or escape detection and elimination by the immune system, has been highlighted as one the emerging hallmarks of cancer, necessary for tumor progression and metastasis [9]. Furthermore, tumors are able to modify anti-tumor immunological reactions, a development known as immunoediting [10]. Several T-cell phenotypes and functional markers have been investigated as potential prognostic and predictive factors in breast cancer, such as CD8+ T-cells [11, 12], FoxP3 [13, 14] and the ζ-chain of the T-cell receptor (see Additional file 1: Table S1) [15–17].

Based on tumor infiltration by T-cells, at least two prognostic scoring systems with promising clinical potential have been developed this far in other types of cancer. Firstly, Brandwein-Gensler includes the lymphocytic host response (LHR) in a risk model for head and neck squamous cell cancer [18]. This model correlates with disease progression and survival, even after adjustment for clinical confounders, and a higher LHR has been associated with increased time to disease progression. Secondly, the ‘Immunoscore’ assesses the density of CD3+ and CD8+ T-cells in the tumor center (TC) and the invasive margin (IM). It was recently shown to outperform the TNM system’s prognostic power for early-stage colorectal cancer [19, 20].

Conflicting evidence exists regarding the relative importance and significance of different T-cell phenotypes, and it is likely that their importance and predictive capacity varies depending on breast cancer subtype and tumor microenvironment. Therefore, it is of considerable scientific interest to study T-cell infiltration in the context of molecular subtypes.

## Methods

A prospective study population of breast cancer patients (n = 43) consecutively operated at the Department of Breast and Endocrine Surgery, Karolinska University Hospital, was enrolled in 2009–2010 for immunological analysis. In order to increase the study population, additional patients were identified from the prospective operation log database during the same time period, operated in a consecutive manner. Thus, the study population consisted of 177 previously untreated patients with primary, unilateral invasive breast cancer without clinical signs of regional or distant metastasis. All patients were scheduled for sentinel lymph node biopsy and received adjuvant treatment according to current treatment protocols.

Clinical parameters, as well as patient and tumor characteristics: i.e. age, tumor size, nodal status, TNM-stage, estrogen and progesterone receptor status, Her2/Neu-status, Ki-67 proliferative index, histological grade, histological subtype, LVI, adjuvant treatment, relapse and mortality was compiled from medical files. For subdivision into intrinsic molecular subtypes, the clinical surrogate parameters of estrogen and progesterone receptor positivity, Her2/Neu-status, and Ki-67 labeling index were applied as described in the St Gallen consensus report from 2013 [5]. Tumors were thus subdivided into the five categories luminal A, luminal B, luminal B/Her2/Neu-overexpressing, non-luminal Her2/Neu-overexpressing and basal-like (triple-negative).

Whole tissue sections of 177 tumors and their corresponding sentinel nodes were retrospectively collected from the Department of Pathology at Karolinska University Hospital for immunological analysis. Before staining, 4 µm thick sections were cut from paraffin-embedded tumor blocks, dewaxed in xylene and rehydrated in decreasing ethanol concentrations, from 100 to 70 %. Pretreatment with citrate buffer (pH 6.0) was performed. Subsequently, samples were heated 10–12 min in a microwave at 750 W up to 98 °C. To facilitate antigen retrieval, samples were further boiled for 20 min at 350 W. Samples were then cooled in a water bath for 20 min. Sections were incubated in 0.5 % H_2_O_2_ for 30 min to prevent endogenous peroxidase activity and thereafter washed in water followed by TBS (Triss-Buffer-Saline). After these initial steps, two different protocols ensued for CD3, ζ-chain- and CD8, FoxP3-staining, respectively.

### CD3 and ζ-chain-staining

Unspecific binding sites were blocked using 1 % BSA (bovine serum albumin) in TBS for 30 min. Without prior washing, the primary antibody diluted in 1 % BSA was dripped on and the samples were incubated in a refrigerator overnight. For CD3, the primary antibody was rabbit-anti-human, Dako (A0452), 1:800. For the ζ-chain, the primary antibody used was mouse-anti-human, Santa Cruz Biotechnology (sc1239), 1:4 000. The next day, samples were taken out from the refrigerator and washed in TBS 3 × 5 min. A biotinylated secondary antibody (Vector Laboratories, 1:200, goat-anti-rabbit, BA-1000 for CD3 and horse-anti-mouse, BA-2000 for CD3-ζ) in 0.2 % Triton-X in TBS was applied, and sections were incubated for 30 min. Meanwhile, the tertiary antibody (ABC-Po, PK-6100, Vector Laboratories) was prepared using two units of Avidin, two units of Biotin and 96 units of 0.2 % Triton-X in TBS. The mix was left in room temperature for 30 min. After the secondary antibody had been applied for 30 min, washing in TBS for 3 × 5 min ensued. The tertiary antibody was applied 30 min after being mixed and sections were once again incubated for 30 min, followed by another washing in TBS for 3 × 5 min. Then DAB-kit, SK-4100 (Vector Laboratories) was used in accordance with the manufacturer’s description. Nuclear staining was done using Mayers HTX (Hematoxylin) and sections were washed in lukewarm water. Sections were then dehydrated, and bathed for a few minutes in increasing concentrations of ethanol, from 70 to 100 %. Finally, they were bathed in xylene and mounted on a xylene-based assemblage-material.

### FoxP3/CD8

Unspecific binding sites were blocked using 2.5 % horse-serum for 30 min. No washing was performed. Primary antibodies diluted in 1 % BSA [FoxP3: mouse-anti-human, eBioscience (14-4777-82), 1:100 and CD 8: rabbit-anti-human, Spring, SP Clone (M3162), 1:100] were mixed into the solution and incubated overnight in a refrigerator-tempered moisture chamber. The next day, sections were washed in TBS 3 × 5 min. Secondary antibodies [FoxP3: biotinylated horse-anti-mouse, Vector Laboratories (BA-2000) and CD8: ImPress peroxidase, anti-rabbit, Vector Laboratories (MP-7401)] were added and then incubated for 30 min. During the incubation time, the tertiary antibody (ABC-AP, AK 5000, Vector Laboraties) was prepared using one unit of Avidin, one unit of Biotin and 98 units of 0.2 % Triton X in TBS. After the secondary antibody had been applied for 30 min, washing in TBS for 3 × 5 min ensued. The tertiary antibody was applied 30 min after being mixed and sections were once again incubated for 30 min, followed by another washing in TBS for 3 × 5 min. Vector Blue Substrate Kit, Vector Laboratories (SK-5300), was used in accordance with the manufacturer’s instructions, followed by addition of DAB-kit, Vector Laboratories (SK-4100). Nuclear staining was done using Mayers HTX (Hematoxylin) and sections were washed in lukewarm water. Finally, sections were mounted on water-based assemblage material.

### Evaluation of occurrence and distribution of CD3, ζ-chain, CD8 and FoxP3

Samples were analyzed by an experienced pathologist together with the student. Patient identification was coded to ensure a blinded assessment. Three areas of interest were selected: the tumor invasive margin, where the front of the tumor cells interact with the surrounding tumor stroma, the tumor center, and the paracortex of the sentinel node. Here, in the main T-cell area of the lymph node, T-cells encounter antigen-presenting cells deriving from the tumor and T-cell activation takes place. The pathologist assessed these three areas of interest in order to identify the high power field (HPF) per area showing the largest amount of T-cells expressing the respective marker for subsequent manual counting. For analysis of the number of cells expressing the marker of interest, both investigators analyzed samples independently of one another. Inter-individual accordance was assessed using approximately 20 samples and deemed to be sufficient, although no statistical analysis was performed to assess a kappa-value. The software ImageJ (http://www.imagej.nih.gov/ij/), which allows the user to mark already counted cells, was used to aid manual cell counting. Cells were counted in the selected HPF with the densest T-cell infiltration of the respective phenotype.

### Assessment of lymphocytic host reaction

LHR was investigated in tissue sections using the definitions provided by Brandwein-Gensler et al. [18].

### Statistical analysis

All distributions were tested for normality using the Shapiro–Wilks test (if n < 50) or the Kolmogorov–Smirnov test (if n > 50) and parametric or non-parametric tests were used accordingly. The Kruskal–Wallis test was used to assess differences in median expression between more than two groups in order to decrease the probability of type 1-errors. If the significance level was found to be below 0.05, the Mann–Whitney U test was used to test any difference between two separate groups specifically. When different areas were compared within the same individuals, Wilcoxon sum rank test for paired samples was used. Occasionally (when normality could not be rejected), Student’s t test and Levene’s test for equality of variance were used for the same purpose. Chi square cross-tabulation was used to test associations between two ordinal or nominal variables. Fisher’s exact test was used for equivalent purposes when the underlying assumptions of Chi Square cross-tabulation were not fulfilled. Bivariate correlations were tested using Pearson Correlation coefficient or Spearman’s Rank Correlation Coefficient depending on data normality. All statistical analyses were performed using IBM SPSS Statistics, version 22.0 (SPSS Inc., Chicago, Illinois, USA). A two-tailed α < 0.05 was considered statistically significant.

## Results

Overall, 177 patients were included. Patient and tumor characteristics are presented in Table 1. Median follow-up time was 47 months (0–58). Seven patients died, three of whose deaths were due to breast cancer. Recurrence was diagnosed in 12 patients, most commonly (seven patients) at a distant location. Local (within the same breast) and axillary recurrence occurred in three patients, respectively. One patient had both axillary and distant recurrences. Due to the low number of events, we do not report on survival analyses; these were performed but shown to not render significance even for established clinical prognostic factors, confirming the unfeasibility of survival analysis in this cohort thus far.

**Table 1:** Patient and tumor characteristics

	N (%)
Age at operation (years)^a^	62 (32–91)
Tumor size (mm)^a^	17.5 (5–100)
*Nodal status*	
N0	124 (70.1)
N1–3	53 (29.9)
*TNM-stage*	
I	85 (48.0)
II	80 (45.2)
III	12 (6.8)
*Estrogen receptor status*	
Negative	26 (14.7)
Positive	151 (85.3)
*Progesterone receptor status*	
Negative	47 (26.6)
Positive	130 (73.4)
*Her2/Neu status (FISH)*	
Negative	154 (87.0)
Positive	23 (13.0)
Ki-67 (%)^b^	18 (1-95)
Low-proliferative	94 (53.1)
High-proliferative	82 (46.3)
*Nottingham histological grade*	
I	38 (21.5)
II	84 (47.5)
III	55 (31.1)
*Molecular subtype*	
Luminal A	88 (49.7)
Luminal B	46 (26.0)
Luminal B Her2/Neu-overexpressing	17 (9.6)
Non-luminal Her2/Neu-overexpressing	6 (3.4)
Basal-like	20 (11.3)
*Lymphovascular invasion (LVI)*	
Yes	24 (13.6)
No	133 (75.1)
*Adjuvant radiotherapy*	
Breast or chest wall only	110 (62.2)
Locoregional	33 (18.6)
*Adjuvant chemotherapy*	
Anthracyclines	53 (29.9)
Taxane-based	30 (16.9)
Other	1 (0.0)
*Adjuvant endocrine therapy*	151 (85.3)
*Anti-Her2/Neu therapy (trastuzumab)*	19 (10.7)

Figures are N and (%) unless otherwise stated

^a^Median (range)

^b^The cut-off value being 20 %

### Lymphocytic host response correlates positively with immunological markers and is associated with poor prognostic factors

Lymphocytic host response (LHR), categorized into four groups (negative, weak, moderate and strong) according to the original description [18], correlated positively with the density of all measured immunological markers (Spearman’s Rho 0.368–0.799, each p < 0.01). Tumors displaying higher degrees of LHR had significantly higher histological grades (p = 0.003) and proliferation (p < 0.001), and were negative for ER and PR (p < 0.001). LHR showed no relation to other clinical parameters.

**Fig. 1:**
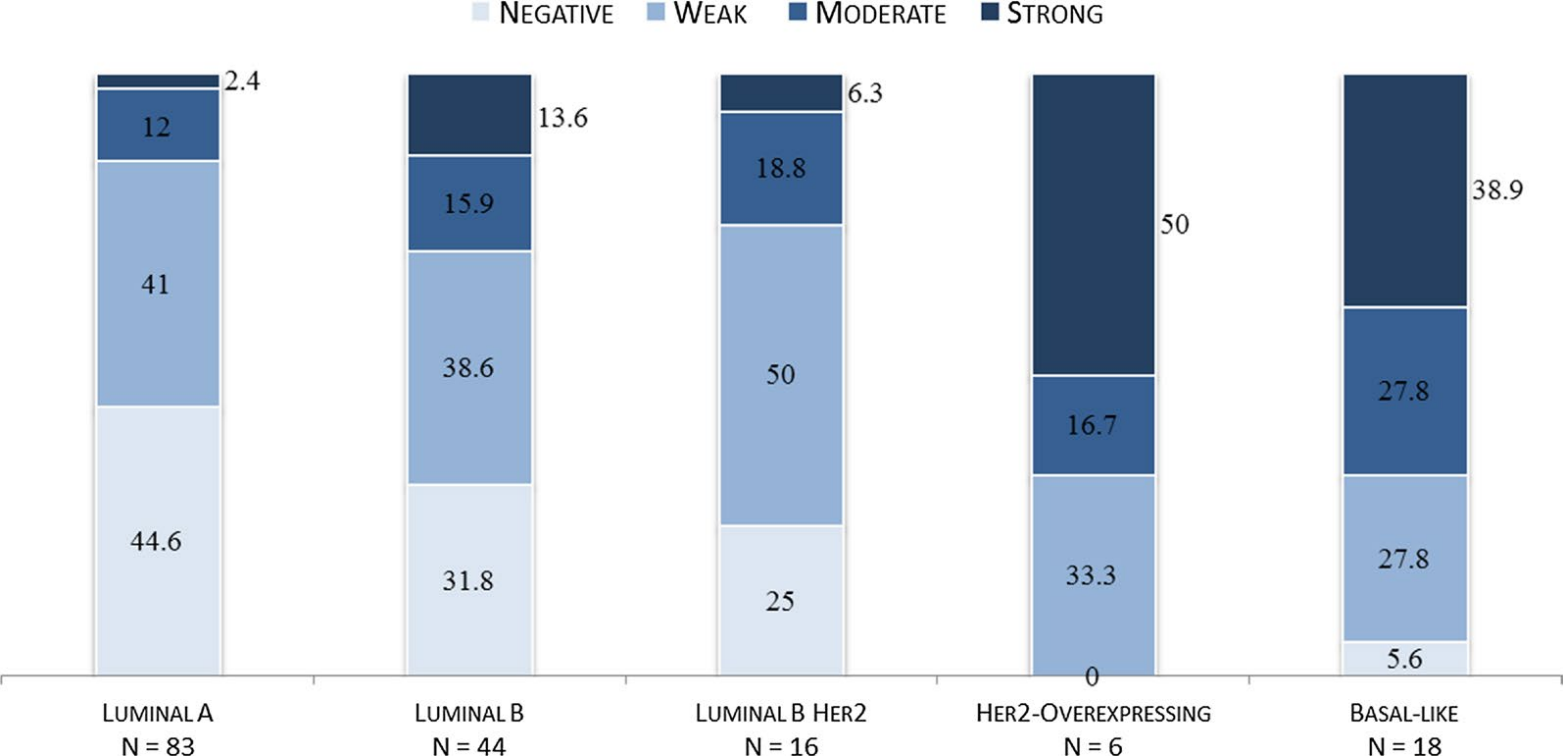
Lymphocytic host response within each molecular subtype: *numbers* represents percentage of *color-coded grades* (negative, weak, moderate, strong) within each tumor subtype

The five intrinsic molecular subtypes (luminal A, luminal B, luminal B Her2/Neu overexpressing, non-luminal Her2/Neu overexpressing and basal-like) differed considerably in their respective LHR (p < 0.001). While the luminal (ER-positive) subtypes generally exhibited a negative or weak LHR, the non-luminal (ER-negative) ones displayed a strong or moderate LHR, as depicted in Fig. 1. In particular, large discrepancies were found for the luminal A subtype against the non-luminal subtypes (p < 0.001).

### The density of T-cell phenotypes differs between tumor areas

**Fig. 2:**
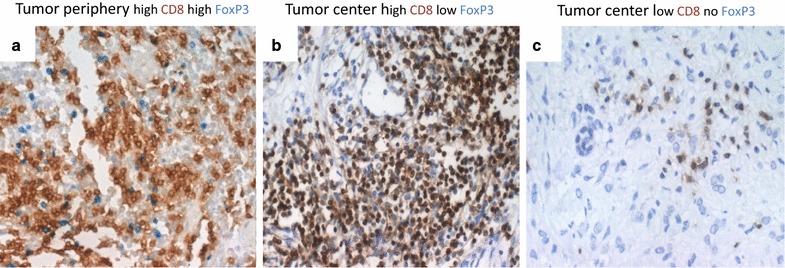
Immunohistochemical staining of whole tumor sections. **a** Tumor periphery displaying high densities of CD8+ (*brown*) and FoxP3+ (*dark blue*) T-cells. **b** Tumor center with high density of CD8+ but few FoxP3+ T-cells. **c** Tumor center with few CD8+ and no FoxP3+ T-cells

To better characterize the immune infiltrate, we performed immunohistochemistry for total T-cells (CD3), cytotoxic T-cells (CD8), regulatory T-cells (FoxP3) and T-cell functionality (CD3-zeta-chain, see Additional file 1: Table S1 for a description of the investigated markers). Representative images for CD8 and FoxP3 are presented in Fig. 2. For all T-cell phenotypes, the mean number of infiltrating cells per high power field (HPF) was significantly higher in the invasive margin (CD3: 306.93 ± 203.32; CD8: 149.37 ± 108.68; FoxP3: 20.26 ± 26.72; CD3-zeta: 98.93 ± 103.09) than in the tumor center (CD3: 126.81 ± 119.35; CD8: 82.84 ± 83.86; FoxP3: 14.90 ± 25.47; CD3-zeta: 31.11 ± 52.32; p < 0.0001). The numbers of positive T-cells in the IM correlated positively with their TC equivalents for all markers (each p < 0.001, Spearman’s Rho 0.415–0.752). Not surprisingly, densities of all analyzed T-cell phenotypes (apart from CD-zeta the density of which was not analyzed in the SLN) were highest in the SLN (CD3: 1528.63 ± 485.81; CD8: 344.14 ± 199.60; FoxP3: 71.66 ± 82.50). In 12 cases, an additional non-sentinel lymph node could be evaluated, but the densities of T-cell phenotypes did not differ from the SLN itself.

### Density of T-cell phenotypes differs significantly in different molecular subtypes

**Fig. 3:**
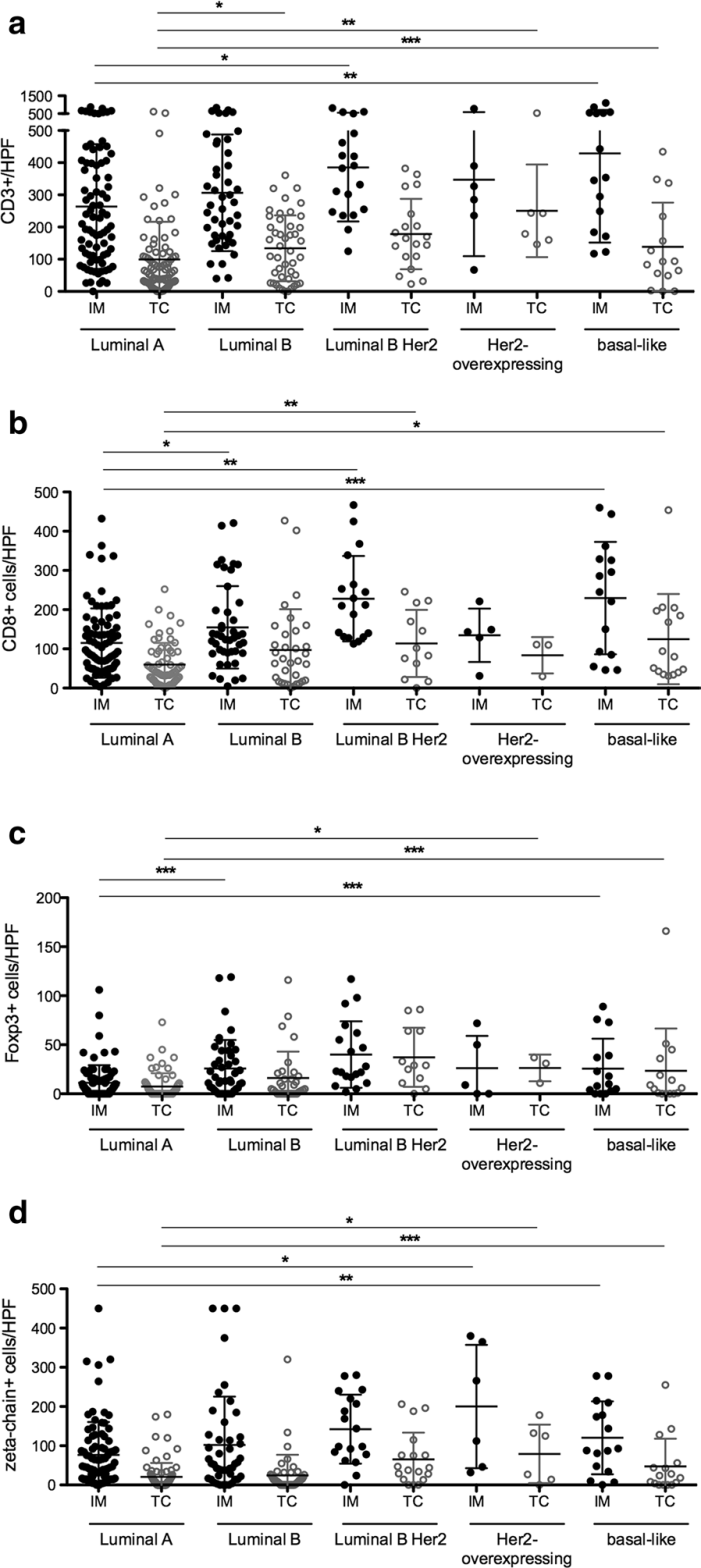
Numbers of T-cells per high power field (HPF) within each tumor molecular subtype and tumor area. **a** CD3, **b** CD8, **c** FoxP3 and **d** CD3-ζ. Significant subgroup differences with the luminal A subtype as reference category are highlighted with *p < 0.05; **p < 0.01 and ***p < 0.001

Overall, all studied T-cell phenotypes differed significantly between molecular subtypes, both in the invasive margin (CD3: p = 0.013; CD8: p < 0.001; FoxP3: p < 0.001 and CD3-ζ: p < 0.007) and the tumor center (CD3: p = 0.001; CD8: p = 0.042; FoxP3: p < 0.001 and CD3-ζ: p = 0.002). The density of all T-cell phenotypes in both invasive margin and tumor center was consistently lowest in the luminal A subtype. Interestingly, the more aggressive subtypes each had a distinct profile, with basal-like tumors showing the highest FoxP3 and CD8 densities, Her2/neu overexpressing tumors the highest CD3-ζ density and luminal B Her2/neu positive tumors the highest CD3 density in the invasive margin and CD8 levels similar to basal-like tumors. All 19 basal-like tumors had some degree of FoxP3 positivity while 29 of 81 (36 %) luminal A-tumors were entirely negative for FoxP3. Figure 3 shows numbers of positive T-cells for each marker per subtype and tumor area, highlighting significant differences between subtypes with the luminal A category as reference group.

A common clinical classification is to separate luminal (ER-positive) tumors from non-luminal ones (ER-negative). Comparing these two groups, all densities of T-cell phenotypes were significantly higher in the non-luminal subtypes (p < 0.001) apart from CD8 density in the tumor center.

The number of T-cell phenotypes in the SLN and non-SLNs was not significantly different when comparing molecular subtypes or luminal versus non-luminal tumors.

### T-cell functionality differs among subtypes

In order to assess the functionality of the T-cells within different subtypes, the number of ζ-chain+ T-cells in relation to CD3+ T-cells was calculated as a ratio. Interestingly, this ratio did not differ significantly between the different subtypes, however, when comparing luminal versus non-luminal tumors, the latter has a significantly lower ratio in the tumor center (p = 0.012) but a higher ratio in the invasive margin (p = 0.022). The ζ-chain/CD3-ratio positively correlated with LHR (Spearman’s Rho 0.581, p < 0.01).

The suppressive versus cytotoxic equilibrium was analyzed using a ratio of FoxP3+ to CD8+ , which differed significantly when comparing all subtypes in both invasive margin and tumor center (p = 0.003). This ratio was consistently lower in the invasive margin than in the tumor center. The highest ratio was found in basal-like tumors (tumor center 0.62 ± 0.64 and invasive margin 0.19 ± 0.15) and the lowest in luminal A tumors (tumor center 0.15 ± 0.29 and invasive margin 0.09 ± 0.12). Here, too, the luminal tumors generally had lower ratios than their non-luminal counterparts, especially in the tumor center (p < 0.001). Finally, the cytotoxic potential of the T-cells was assessed by creating a ratio of CD8+ to CD3+ T-cells. This ratio did not differ significantly between subtypes. No differences between subtypes were found in SLNs.

### T-cell phenotypes in the tumor and molecular subtypes are related to established clinical parameters

Higher densities of T-cell phenotypes were invariably associated with negative prognostic factors as measured by routine histopathological assessment. This held true for all markers with significances listed in Table 2. Interestingly, a higher density of CD8+ and CD3+ T-cells in the paracortex of the SLN was associated with the debated prognostic factor of lymphovascular tumor invasion (LVI). A higher ζ-chain/CD3 ratio was associated with ER (p = 0.022 and 0.012 for invasive margin and tumor center), PR negativity (p = 0.003 for tumor center), and ductal tumor histology. While the CD8/CD3 ratio was higher in the SLN of younger individuals, it was also higher in the tumor center of patients 50 years of age or older. A higher FoxP3/CD8 ratio was significantly associated with ER negativity (p = 0.031 and <0.001 for invasive margin and tumor center), high proliferation (both areas p < 0.001) and tumor histological grade (p = 0.013 and 0.034, respectively).

**Table 2:** Density of T-cell phenotypes in relation to established prognostic markers

	CD8	FoxP3	ζ-chain	CD3
*Invasive margin*				
Age at operation (<50 vs ≥50 years)	n.s.	n.s.	n.s.	n.s.
Tumor size (T1 vs T2 vs T3)	n.s.	n.s.	n.s.	n.s.
Nodal status (N0 vs N1)	n.s.	n.s.	n.s.	n.s.
Tumor stage (TNM)	n.s.	n.s.	n.s.	p = 0.027
Estrogen receptor status (pos. vs neg.)	n.s.	p = 0.001	p = 0.001	p = 0.025
Progesterone receptor status (pos. vs neg.)	p = 0.004	p = 0.044	p = 0.001	p = 0.002
Her2/Neu status (FISH) (amplified/not amplified)	p = 0.038	n.s.	p = 0.039	n.s.
Proliferation (Ki-67) (high vs low)	p = 0.001	p < 0.001	p = 0.024	p = 0.027
Nottingham histological grade	p < 0.001	p < 0.001	p = 0.051	p = 0.030
Histological subtype (ductal vs lobular)	n.s.	n.s.	p = 0.030	n.s.
Lymphovascular invasion (pos. vs neg.)	n.s.	n.s.	n.s.	n.s.
*Tumor center*				
Age at operation (<50 vs ≥50 years)	n.s.	n.s.	n.s.	p < 0.001
Tumor size (T1 vs T2 vs T3)	p = 0.009	n.s.	n.s.	p = 0.001
Nodal status (N0 vs N1)	n.s.	n.s.	n.s.	n.s.
Tumor stage (TNM)	p = 0.010	p = 0.050	n.s.	p = 0.001
Estrogen receptor status (pos. vs neg.)	p = 0.001	p < 0.001	p < 0.001	p < 0.001
Progesterone receptor status (pos. vs neg.)	p = 0.001	p = 0.009	p < 0.001	p = 0.001
Her2/Neu status (FISH) (amplified/not amplified)	n.s.	n.s.	n.s.	p = 0.040
Proliferation (Ki-67) (high vs low)	p = 0.045	p < 0.001	p < 0.001	p < 0.001
Nottingham histological grade	p = 0.002	p = 0.005	p = 0.009	p = 0.001
Histological subtype (ductal vs lobular)	n.s.	n.s.	n.s.	n.s.
Lymphovascular invasion (pos. vs neg.)	n.s.	n.s.	n.s.	n.s.
*Sentinel node paracortex*				
Age at operation (<50 vs ≥50 years)	p < 0.001	n.s.	n.s.	n.s.
Tumor size (T1 vs T2 vs T3)	n.s.	n.s.	n.s.	n.s.
Nodal status (N0 vs N1)	n.s.	n.s.	n.s.	n.s.
Tumor stage (TNM)	n.s.	n.s.	n.s.	n.s.
Estrogen receptor status (pos. vs neg.)	n.s.	n.s.	n.s.	n.s.
Progesterone receptor status (pos. vs neg.)	n.s.	n.s.	n.s.	n.s.
Her2/Neu status (FISH) (amplified/not amplified)	p = 0.022	n.s.	n.s.	n.s.
Proliferation (Ki-67) (high vs low)	p = 0.021	n.s.	n.s.	n.s.
Nottingham histological grade	n.s.	n.s.	n.s.	n.s.
Histological subtype (ductal vs lobular)	n.s.	n.s.	n.s.	n.s.
Lymphovascular invasion (pos. vs neg.)	p = 0.032	n.s.	n.s.	p = 0.006

Significant values always represent a higher T-cell infiltration for any negative prognostic group

Molecular subtypes were confirmed to be related to established clinical parameters: while 40 % of all basal-like tumors affected patients below the age of 50, all tumors of the luminal B Her2/Neu-positive subtype were found in patients above 50 years of age. Luminal A tumors also predominantly affected the older age group, in 83.9 % of cases. Non-luminal tumors were associated with higher histological grades. Lymphovascular invasion (LVI) was most commonly observed in the luminal B Her2/Neu-positive subtype (35.7 %), and most infrequently in luminal A tumors (7.7 %). No significant association, however, was found regarding tumor size, nodal status, TNM stage or disease relapse.

### An immunological score based on CD3 and CD8 separates molecular subtypes

In order to develop a scoring system based on immunological markers that could accurately separate the molecular subtypes from one another, the density of CD8+ and CD3+ T-cells in both tumor areas was compounded and categorized as either high or low. Samples were excluded if the value of either CD8 or CD3 was missing. Samples were divided into three groups: score I, with tumors exhibiting low density of both markers; score II, tumors displaying high density of either marker (in CD3^high^ but CD8^low^ cases most likely CD4-dominated tumors) and score III, tumors with high density of both T-cell phenotypes. CD3 and CD8 were selected in accordance with the immunological score for colorectal cancer described earlier [20].

**Fig. 4:**
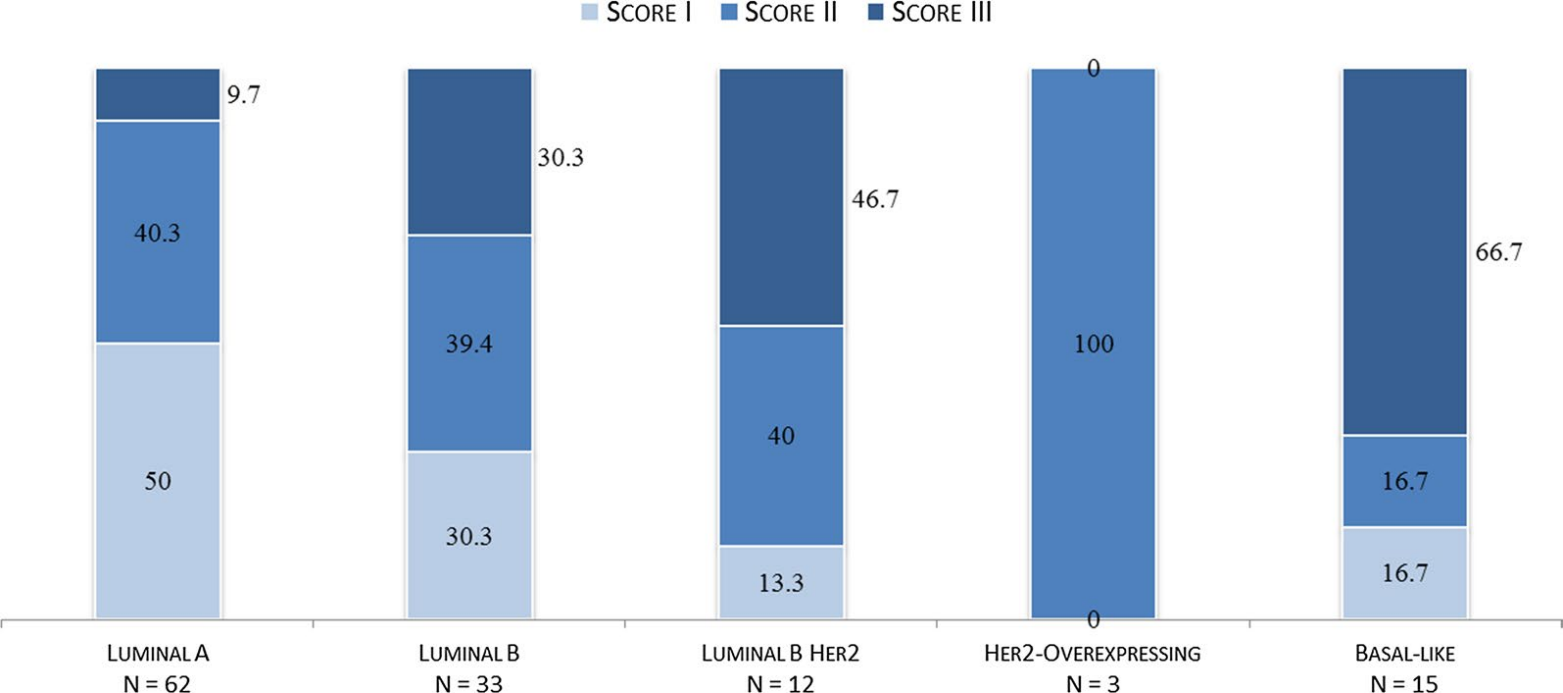
Distributions of immunological scores within each subtype. *Numbers* represent the percentage of each score (I–III) within each molecular subtype

In total, 125 samples could be assessed. Tumors displayed substantially different immunological scores among the molecular subtypes (p < 0.001), with basal-like tumors predominantly showing a high, and luminal A tumors a low score. Figure 4 illustrates the percentage of tumors within each immunological score for each molecular subtype.

## Discussion

This report describes differential densities of T-cells expressing the markers CD8, FoxP3, ζ-chain and CD3 in different molecular subtypes of breast cancer and their sentinel lymph nodes. We have here shown that molecular subtypes exhibit different densities of all analyzed T-cell phenotypes and that higher densities are associated with more aggressive tumor subtypes. We could further show that an immunological score based on CD3+ and CD8+ T-cells markedly differed between tumor subtypes.

The presence of tumor-infiltrating lymphocytes has repeatedly been shown to correlate with breast cancer prognosis and treatment response [11–16]. One of the here applied parameters, the lymphocytic host response (LHR) as described by Brandwein-Gensler [18], takes all lymphocytes into account and is based on routine hematoxylin-eosin staining, while all other parameters are specific T-cell markers detected by immunohistochemistry. Interestingly, the overall lymphocytic response yielded very similar results to the more sophisticated immunohistochemical analysis, despite the notion of functional heterogeneity in tumor-infiltrating lymphocytes: no marker had any superior or inferior capacity to illustrate differences among subtypes. Even though we could show that expression of FoxP3 seems to be a specific feature of basal-like tumors, our results suggest that the overall T-cell infiltration, rather than infiltration of specific phenotypes, separates the molecular subtypes. Luminal (ER+) subtypes were denoted by a low immunological response while non-luminal (ER−) subtypes showed clear signs of an immunological host response. The luminal A subtype in particular distinguished itself with a low immunological response: only 2.4 % of all luminal A tumors had a strong LHR, in contrast to 41.7 % of the non-luminal subtypes. These findings are in support of several reports of non-luminal tumors generally exhibiting a higher degree of tumor-infiltration by lymphocytes [21, 22], even though there are contrary results, too [23]. The interesting question is why differential immune infiltration should occur in breast cancer, and how it is associated with prognosis. It seems feasible to assume that the infiltration of immune cells should reflect underlying biological properties of the tumor [20]. Differential gene expression, on which the distinction of molecular subtypes is based, may cause differences in vascularization, lymphatic vessel density, antigenicity, chemokine milieu or cytokines. The occurrence of high endothelial venules, for example, is associated with lymphocytic infiltration and corresponds to a more favorable prognosis in breast cancer, however, an association with molecular subtypes has not yet been explored [24]. Cytotoxic T-cell responses are increasingly found to be directed against mutated epitopes predicted to be immunogenic, and are associated with better patient survival [25]. These inherent distinctions may explain why the subtypes, originally defined by dissimilar gene expression, exhibit inherently differential densities of T-cells. We have previously shown that signs of immunological dysfunction can be detected in the tumor, and even in peripheral blood, of patients with early-stage disease and limited tumor burden [17, 26]. While it became aggravated with tumor progression, these studies suggest that the immune environment is shaped relatively early during breast cancer development.

Interestingly, subtypes associated with a poor prognosis generally had higher numbers of each T-cell phenotype, including those phenotypes that should imply a favorable prognosis, such as CD8+ cytotoxic T cells. While it seems somewhat counter-intuitive at first glance, the higher degree of lymphocytic response, however, predicts an improved response to both adjuvant and neoadjuvant treatment [7, 14, 27]. Consequently, each tumor subtype probably has an inherent degree of infiltration of T-cells, and any prognostic deductions should therefore be done with these inherent differences in mind. Especially in basal-like and Her2/Neu-overexpressing breast cancer, it has been suggested that a subdivision in tumors with high and low levels of tumor-infiltrating lymphocytes should be attempted for potentially adoptive treatment [28].

In addition, our results suggest that some subtypes might be more likely to respond to immunotherapeutic approaches than others. For example, a first clinical trial targeting the checkpoint inhibitory molecule PD1 showed promising results in patients with metastatic triple-negative tumors [29]. In our study the related basal-like subtype had the strongest degree of immune infiltration, in agreement with studies showing that basal-like tumors are the subtype most frequently exhibiting PD-L1-positive tumor cells as well as immune infiltrate, which correlated with T-cell infiltration and a better prognosis [30]. Of note, some studies found PD-L1 to correlate with poor survival [31]. This likely reflects omission of subtype-specific analysis and/or failure to detect PD-L1 expression, which is IFNγ dependent, in poorly infiltrated subtypes. Such discrepancies illustrate the importance of taking into account the differential immune infiltration patterns within the cancer subtypes.

Another important finding is the fact that all tumor subtypes generally exhibited a more pronounced infiltration in the invasive margin than in the tumor center. This is in agreement with findings from other tumor entities that show that it can be difficult for T-cells to infiltrate into tumors, especially those with a large stromal component, due to physical barriers or the hostile tumor microenvironment [32]. Interestingly, high T-cell densities in both the invasive margin and the tumor center predict better survival in colorectal cancer patients, where a ‘tumor-observing’ immune response around the invasive margin is commonly found [33]. Importantly, we also observed a difference in the proportion of immune cells present at different sites: all tumor types showed a higher ratio of FoxP3+ cells to CD8+ cells in the tumor center, which is coherent with reports showing that tumors can recruit regulatory T-cells as part of their immune escape mechanism, and that these are better adapted to the tumor microenvironment, as they for example are more resistant to reactive oxygen [34, 35]. This would also be in line with the decreased detection of the CD3-zeta chain in the tumor center, as this molecule is known to be down-regulated under conditions of oxidative stress.

To the best of our knowledge, no study has yet evaluated the relationship between T-cell densities in breast cancer sentinel lymph nodes and molecular subtypes. Compared to our observations in tumor tissue, sentinel lymph node analysis yielded far fewer inter-subtype differences. Our study population, however, consisted of clinically node-negative patients, and in 70 % of patients, node negativity was confirmed by histopathology. Some other studies have seen associations between increased FoxP3 expression in sentinel nodes and nodal metastasis [36], while others did not [37]. Considering that different subtypes have different propensities of developing nodal metastasis [38], differential FoxP3 expression could possibly have been detected had the study included later-stage patients. The only marker which differed significantly among molecular subtypes in the sentinel node was CD8, with higher densities being seen in the luminal B Her2 positive-subtype compared to luminal A tumors. This may reflect the general immunogenicity of this tumor subtype, as luminal B Her2 tumors also had the highest number of tumor-infiltrating CD8+ T-cells. Similarly, this subtype had the highest degree of LVI, which may explain the association with CD8+ accumulation in the sentinel node.

The immunoscore developed for colorectal cancer predicts relapses among early-stage tumors more accurately than classical TNM staging and consequentially bears therapeutic implications; patients at high risk could potentially benefit from adjuvant treatment not normally given in early-stage disease [19, 20]. De Kruijf et al. [39] used a panel of immunological markers to construct seven immune subtypes in early breast cancer based on biological rationale. After an initial training set, these immune subtypes differed significantly in a multivariate analysis for relapse-free time in an independent validation set. These results indicate that using immunological markers for risk stratification purposes has potential also in the setting of breast cancer. Analogously, we have here shown that a scoring system based on densities of CD3+ and CD8+ T-cells significantly distinguishes molecular subtypes. Since the subtypes predict prognosis, and densities of immunological markers are known to do likewise, further research should compare long-term survival and relapse data among these different immunological scores, potentially separating high-risk patients within each subtype.

## Conclusions

Molecular subtypes of breast cancer exhibit differential tumoral densities of CD8+ , FoxP3+ , ζ–chain+ and CD3+ T-cells. Large variations were observed within each tumor subtype, especially in quantity, rather than functionality of T-cells. This may have prognostic implications but needs to be validated using a larger patient population with longer follow-up time.

## Additional file

10.1186/s12967-016-0983-9 Phenotypic and functional markers used for analysis of T-cell infiltrates.

## Abbreviations

CD: cluster of differentiation
ER: estrogen receptor
FISH: fluorescent in situ hybridization
FoxP3: forkhead box P3
Her2/neu: human epidermal growth factor receptor–2
HPF: high-power field
IM: invasive margin
LHR: lymphocytic host response
LVI: lymphovascular invasion
PR: progesterone receptor
TIL: tumor-infiltrating lymphocytes
TC: Tumor Center
TNM: tumor, node, metastasis
