# Supplementary material for: Differential tumor infiltration by T-cells characterizes intrinsic molecular subtypes in breast cancer
Source: J Transl Med. 2016 Jul 29;14:227. doi: 10.1186/s12967-016-0983-9 (PMC4966793; doi:10.1186/s12967-016-0983-9)
Supplement: Supplementary file 1 — 10.1186/s12967-016-0983-9 Phenotypic and functional markers used for analysis of T-cell infiltrates. [file 12967_2016_983_MOESM1_ESM.docx]

**Supplemental Table 1: Phenotypic and functional markers used for analysis of T-cell infiltrates.**

| **Marker** | **Antibody** | **Function** |
| --- | --- | --- |
| CD3 | Polyclonal rabbit anti human | CD3 is part of the T cell receptor complex, expressed on all mature T lymphocyte and thus used as a T cell marker. It contributes to antigen recognition, signal transduction and thus T cell activation. The immunogen recognized by the antibody used is part of the CD3epsilon chain. |
| CD8 | Rabbit monoclonal anti human, clone SP16 | CD8 is a co-receptor of MHC I binding by T cell receptors and is used as a marker to identify cytotoxic T lymphocytes. |
| FoxP3 | Mouse monoclonal anti human, clone 236A/E7 | Forkhead box P3 (FoxP3) protein is a transcription factor used to define regulatory T cells, as it is constitutively expressed by CD4+ natural regulatory T cells. Absence of FoxP3 is associated with severe autoimmune pathology. |
| CD3-ζ | Mouse monoclonal anti human, clone 6B10.2 | The CD3 ζ -chain forms part of the T-cell receptor complex. Receptor engagement leads to ζ-chain dimerization, which relays activating signals via phosphorylation of the ζ-chains ITAM motifs, which in turn regulate activity of protein kinases such as ZAP-70. |
